# HIV and syphilis testing preferences among men who have sex with men and among transgender women in Lima, Peru

**DOI:** 10.1371/journal.pone.0206204

**Published:** 2018-10-29

**Authors:** Claire C. Bristow, Noah Kojima, Sung-Jae Lee, Segundo R. Leon, Lourdes B. Ramos, Kelika A. Konda, Brandon Brown, Carlos F. Caceres, Jeffrey D. Klausner

**Affiliations:** 1 Department of Medicine, University of California San Diego, La Jolla, CA, United States of America; 2 David Geffen School of Medicine, University of California Los Angeles, Los Angeles, CA, United States of America; 3 School of Public Health, Universidad Peruana Cayetano Heredia, Lima, Peru; 4 Department of Global Health, University of Washington, Seattle, WA, United States of America; 5 Department of Medicine, University of California Riverside, Riverside, CA, United States of America; Hunter College, UNITED STATES

## Abstract

**Background:**

Men who have sex with men (MSM) and transgender women in Peru are at high risk for acquiring syphilis and HIV infection. The World Health Organization highly recommends screening for HIV and syphilis to reduce morbidity and mortality associated with untreated infections. We aimed to identify factors associated with dual testing preferences for HIV and syphilis infection among MSM and transgender women in Lima, Peru.

**Methods:**

We used conjoint analysis, an innovative method for systematically estimating consumer preferences. We created eight hypothetical test profiles varying across six dichotomous attributes: cost (free vs. $4), potential for false positive syphilis result (no false positive vs. some risk of false positive), time-to-result (20 minutes vs. 1 week), blood draw method (finger prick vs. venipuncture), test type (rapid vs. laboratory), and number of draws (1 vs. 2). We fit a conjoint analysis model for each participant using a simple main effects ANOVA. Attribute importance values were calculated using percentages from relative ranges in the attribute’s utility values. Results were summarized across participants and averages were reported.

**Results:**

We recruited 415 MSM/transgender women over 18 years of age from two STD clinics in Lima, Peru. No potential for syphilis false positive result (no false positive vs. some potential for false positive) had the largest average impact on willingness to use the test and on average accounted for 23.8% of test type preference, followed by cost (free vs. ~USD$4; 21.6%), time to results (20 minutes vs. 1 week; 17.4%), number of blood draws (1 draw vs. 2 draws; 13.8%), method of blood draw (fingerprick vs. venipuncture; 13.7%), and test type (rapid POC vs. laboratory; 9.7%).

**Conclusion:**

MSM/transgender women in Peru prioritized accuracy, cost, timeliness and number of blood draws for HIV and syphilis testing. Implementing a low cost, accurate, rapid and dual testing strategy for HIV and syphilis could improve screening uptake and accessibility of testing to accelerate time to treatment.

## Background

The World Health Organization highly recommends screening for human immunodeficiency virus (HIV) and syphilis to reduce morbidity and mortality associated with untreated infections [1, 2]. In South America, HIV and syphilis is endemic among men who have sex with men (MSM) and transgender women [3–5]. In a study conducted in 2013 to 2014 among MSM and transgender women in Lima, Peru, our group observed the prevalence of HIV infection to be 30.1% among MSM and 33.7% among transgender women and the prevalence of recent syphilis infection to be 16.8% among MSM and 6.7% among transgender women [6]. In addition, MSM and transgender women may be averse to health care engagement and those who access care are often lost to follow up [7–11]. Among MSM and transgender women attending sexually transmitted infection (STI) clinics in Lima, Peru, 41% reported never being tested for HIV prior to their current visit [12].

Implementing a preferred and acceptable dual testing strategy for HIV and syphilis could improve screening uptake, accessibility, retention, and buy-in from patient. For example, the introduction of point-of-care (POC) rapid tests, rapid tests that can be used in settings outside of the laboratory, improved overall rates of syphilis screening and treatment among pregnant women in low-resource countries, including Peru [13–15]. However, many types of diagnostics tools are available for syphilis screening and preferred testing modalities for MSM and transgender women have not been determined in South America [16–18]. These testing tools include rapid tests for HIV and syphilis as well as laboratory based tests.

We aimed to determine which attributes of diagnostic tests are preferred among MSM and transgender women in Lima, Peru.

## Methods

### Participants and study setting

MSM and transgender women aged 18 years and over seeking testing or care in one of two STI clinics in Lima, Peru were recruited to participate in a study to assess HIV and syphilis dual testing acceptability and preferences. The two clinical sites were the Alberto Barton Clinic, a public STI Health Center located in Callao, the main port of Peru that regularly provides services to female and male sex workers, MSM and transgender women; and the Epicentro Clinic, a gay men’s community health center in southern Lima provides health services to MSM and transgender women. All interviews were conducted individually by trained counselors in the two clinics in-person in a private room and responses were collected electronically. Interviews took approximately 15 minutes and participants were reimbursed approximately USD$4 for their time.

### Data collection and procedures

Conjoint analysis is a method for systematically estimating consumer preferences [19], and has been applied in health care research [20, 21]. Therefore, we used conjoint analysis to determine which attributes of diagnostic tests are preferred among MSM and transgender women in Lima, Peru.

We created eight hypothetical test profiles, which varied across six dichotomous testing attributes to yield 64 possible test profiles (2^6^ = 64). Given that the number of possible test profiles is too large to ask participants to rate every scenario, we used a fractional factorial design to reduce the number of hypothetical test profiles to eight to measure the main effect of each attribute [22, 23]. That design with eight hypothetical test profiles is 100% efficient, balanced and orthogonal [23]. The testing attributes were: test type (rapid POC vs. laboratory), cost (free vs. approximately four US dollars in the local currency (Peruvian Nuevos Soles S/.12)) (the price of 1 liter of milk at this time was about S/.3.85–4.16 [24]), potential for false positive syphilis result (no risk of false positive vs. some risk of false positive), time-to-result (20 minutes vs. 1 week), blood draw method (finger prick vs. venipuncture), and number of blood draws (1 vs. 2). We included potential for false positive result because often screening tests that are used for syphilis that detect treponemal antibody, this is an antibody that is produced in response to syphilis infection, however this antibody can be present for life even following curative syphilis treatment. Therefore, if a person has had syphilis in the past, they may still test positive for syphilis using a treponemal test. The testing attributes were created using characteristics of existing HIV and syphilis testing strategies [25–27].

Each participant was presented with the eight different testing scenarios, one at a time, described on laminated cards. The cards were presented in random order and were not marked with any schema that might suggest a sequence or preference rating. Participants were asked to rate the eight testing scenarios in terms of how likely they would be to ever test for HIV and syphilis given the test attributes on that test profile card. Participants’ ratings of each hypothetical test profile were recorded using a five-point Likert preference scale: highly likely, somewhat likely, neutral, somewhat unlikely, and highly unlikely.

### Data analysis

The overall goal of conjoint analysis is to understand preferences between product (in this case, a test for HIV and syphilis) attributes and the impact of individual attributes on consumers’ willingness to use a product. Because it may not be possible for a test for HIV and syphilis to be a combination of every desirable attribute, trade-offs must be made to select those attributes that are most highly valued. For analysis, we converted the Likert preference scale values to a 100-point numeric scale using multiplication; higher scores suggested increased preference. We fit a conjoint analysis model for each participant using a simple main effects ANOVA with attributes as independent variables [28], participant ratings as the dependent variable to give part-worth utility values (represented by the β terms from the ANOVA). Part-worth utilities mean values were calculated from each individuals’ subjective preference or the utility they associated with each level (in this study we had two levels) of each attribute. The part-worth utility values measure how much attribute and level influenced the participants’ willingness to use a test. Participants who gave the same Likert scale response for every hypothetical test profile or whose adjusted R square were less than 0.3 were excluded from data analysis. Attribute importance values were calculated using percentages from relative ranges in the attribute’s utility values. Results were summarized across participants and averages were reported. Data were analyzed using SAS v9.4 (Cary, NC, USA).

### Ethical approval

All participants gave written informed consent for participation. Ethical approval and oversight was provided by the Institutional Review Board at the Universidad Peruana Cayetano Heredia. The approval was filed under the reference number SIDISI 59996.

## Results

We recruited 415 MSM and transgender women, with a mean age of 30.1 years (standard deviation: 9.0). Of the 415 participants, 310 (74.7%) were men and 105 (25.3%) were transgender women.

**Fig 1:**
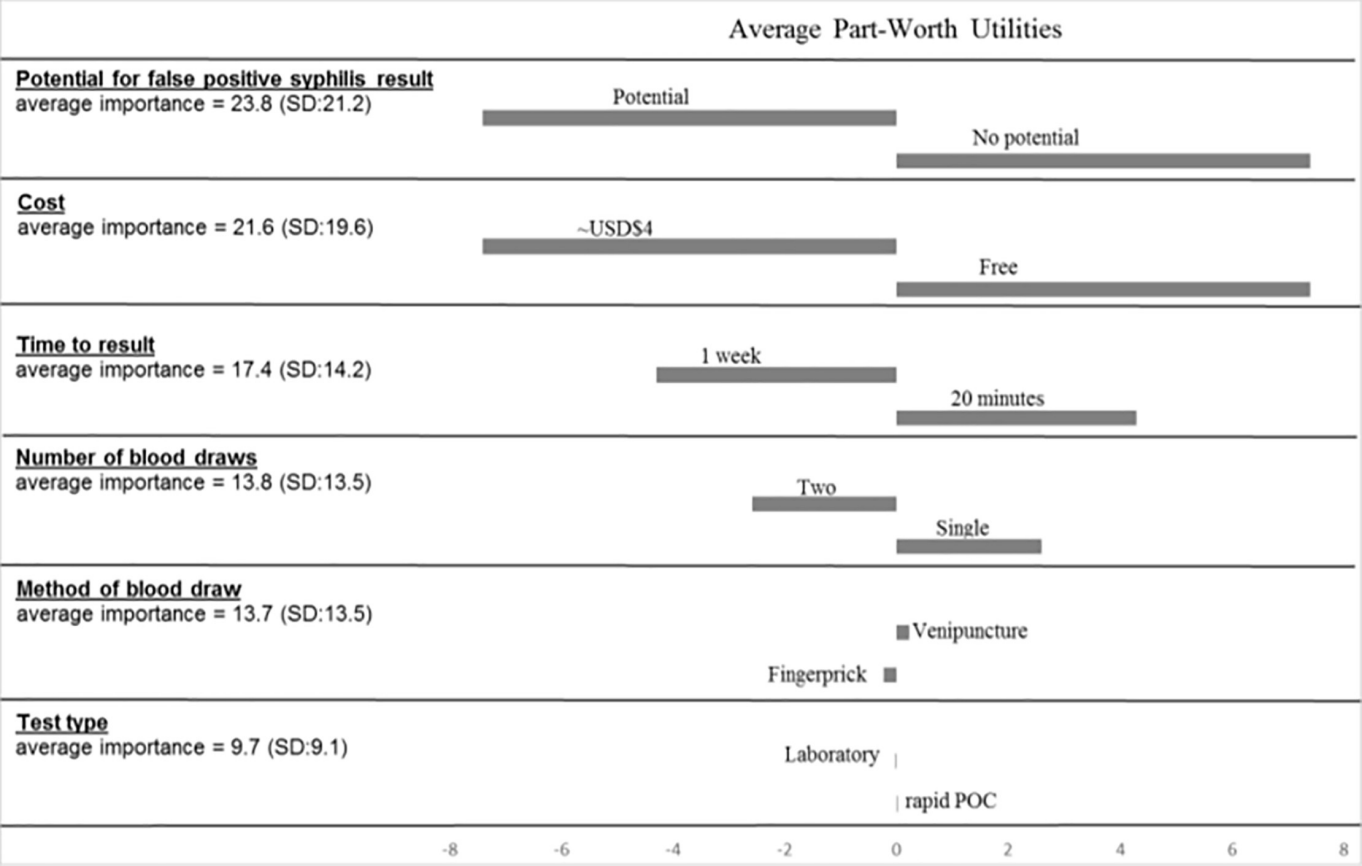
Average importance and average part-worth utilities for attributes of human immunodeficency virus and syphilis testing from conjoint analysis results among men who have sex with men and transgender women in Lima, Peru. *Standard deviation, SD; United States Dollar, USD; Point of Care, POC. Positive values suggest participant relative preference while negative values suggest lack of preference. Part-worth utility values measure how much attribute and level influenced the participants’ willingness to test. Attributes with the largest part-worth utility range are considered the most important in predicting willingness to test. The attribute importance values were calculated using the percentages from relative ranges in the attribute’s utility values.

Of the 415 participants, 40 gave the same Likert scale response for all eight hypothetical test profiles and thus were excluded from the conjoint analysis. In addition, 28 who had an adjusted R square less than 0.3 were excluded from analysis, leaving 347 evaluable results. Average preference scores of the eight hypothetical testing scenarios ranged from 44.4 (SD = 32.0) to 79.2 (SD = 28.2). Results of the metric conjoint analysis model of participant willingness to use a hypothetical HIV and syphilis test are displayed in Fig 1. For the sample population, no potential for syphilis false positive result (no false positive vs. some potential for false positive) had the largest average impact on willingness to use the test and on average accounted for 23.8% of test type preference, followed by cost (free vs. ~USD$4; 21.6%), time to results (20 minutes vs. 1 week; 17.4%), number of blood draws (1 draw vs. 2 draws; 13.8%), method of blood draw (fingerprick vs. venipuncture; 13.7%), and test type (rapid POC vs. laboratory; 9.7%). We stratified the conjoint analysis by gender identity (men vs. transgender women), however we did not observe any significant differences in testing preferences between groups.

## Discussion

We conducted a study among a key group of MSM and transgender women to identify HIV and syphilis testing preferences using metric conjoint analysis. HIV and syphilis testing preferences among those key populations in Peru prioritized accuracy, cost, timeliness, and number of blood draws. While it is well described that MSM and transgender women have difference risk contexts, their preferences regarding HIV and syphilis testing preferences are not dissimilar in our setting. However, given their different risk contexts, it is likely that testing programs will require different strategies to target those groups and further research may be needed to determine those optimal testing programs.

Lee et al. used conjoint analysis to address the willingness to test for HIV among MSM in Los Angeles based on varying factors that found similar results to our study [21]. Populations in both studies prioritized cost and timeliness of results. Among our participants, the method of specimen collection (venipuncture vs. fingerprick) would not affect their likelihood of testing for HIV and syphilis. Additionally, the participants did not prioritize the testing modality, laboratory versus rapid POC. This is unsurprising because this attribute does not provide further information about how the test will be administered and results generated. In addition, participants identified preferred attributes of a POC test that have low potential for a false positive syphilis result, however rapid tests for syphilis usually detect treponemal antibody, thus they cannot distinguish between past and present infection. Rapid tests that include a non-treponemal result, a second test that is often used for confirmation, may have additional utility in this population [29, 30].

There are some potential limitations to this study. The study sample may not be generalizable to all MSM and transgender women in Lima, Peru. The study sample selection also could have impacted the findings in that the participants were already seeking HIV and syphilis testing, therefore attributes of the test would not necessarily have as large of an impact for them compared with those not seeking testing. This may have led to our findings that some attributes of tests were similarly important when deciding whether to test for HIV and syphilis. In addition, further research is needed to determine if availability of preferred tests leads to improved test uptake.

Given that MSM and transgender women are often difficult to access populations that are often lost to follow up, it is important for policy makers and health care workers to understand these groups’ preferences if there is the potential to increase testing and retention in care [7–11]. We found that MSM and transgender women prefer tests that have lower costs, improved accuracy, timeliness, and lower number of blood draws. Policy makers should ensure access to accurate HIV and syphilis tests at a low cost. Test manufacturers should work to create tests that reduce numbers of blood draws and provide accurate results in a timely manner. Health care workers should prioritize optimal clinic management to reduce that chance of having to redraw blood from patients and to provide testing results in a timely manner.

## Supporting information

S1 Dataset PlosOne_dataset_Bristow_conjoint analysis Peru_31aug2018. (XLS)
